# A mock circulation loop to test extracorporeal CO_2_ elimination setups

**DOI:** 10.1186/s40635-020-00341-z

**Published:** 2020-09-11

**Authors:** Leonie S. Schwärzel, Anna M. Jungmann, Nicole Schmoll, Frederik Seiler, Ralf M. Muellenbach, Joachim Schenk, Quoc Thai Dinh, Robert Bals, Philipp M. Lepper, Albert J. Omlor

**Affiliations:** 1 Department of Internal Medicine V - Pneumology and Intensive Care Medicine, University Hospital of Saarland, Kirrbergerstr. 1, 66421 Homburg, Germany; 2 Department of Anaesthesiology and Critical Care, Campus Kassel of the University of Southampton, Kassel, Germany; 3 Department of Transfusion Medicine and Hemostaseology, University Hospital of Saarland, Homburg, Germany

**Keywords:** ECCO_2_R, Mock circulation, ECMO, ARDS, COPD

## Abstract

**Background:**

Extracorporeal carbon dioxide removal (ECCO_2_R) is a promising yet limited researched therapy for hypercapnic respiratory failure in acute respiratory distress syndrome and exacerbated chronic obstructive pulmonary disease. Herein, we describe a new mock circuit that enables experimental ECCO_2_R research without animal models. In a second step, we use this model to investigate three experimental scenarios of ECCO_2_R: (I) the influence of hemoglobin concentration on CO_2_ removal. (II) a potentially portable ECCO_2_R that uses air instead of oxygen, (III) a low-flow ECCO_2_R that achieves effective CO_2_ clearance by recirculation and acidification of the limited blood volume of a small dual lumen cannula (such as a dialysis catheter).

**Results:**

With the presented ECCO_2_R mock, CO_2_ removal rates comparable to previous studies were obtained. The mock works with either fresh porcine blood or diluted expired human packed red blood cells. However, fresh porcine blood was preferred because of better handling and availability. In the second step of this work, hemoglobin concentration was identified as an important factor for CO_2_ removal. In the second scenario, an air-driven ECCO_2_R setup showed only a slightly lower CO_2_ wash-out than the same setup with pure oxygen as sweep gas. In the last scenario, the low-flow ECCO_2_R, the blood flow at the test membrane lung was successfully raised with a recirculation channel without the need to increase cannula flow. Low recirculation ratios resulted in increased efficiency, while high recirculation ratios caused slightly reduced CO_2_ removal rates. Acidification of the CO_2_ depleted blood in the recirculation channel caused an increase in CO_2_ removal rate.

**Conclusions:**

We demonstrate a simple and cost effective, yet powerful, “in-vitro” ECCO_2_R model that can be used as an alternative to animal experiments for many research scenarios. Moreover, in our approach parameters such as hemoglobin level can be modified more easily than in animal models.

## Background

Extracorporeal carbon dioxide elimination (ECCO_2_R) is a method to counteract hypercapnic respiratory failure, e.g., in severe acute respiratory distress syndrome (ARDS). Furthermore, ECCO_2_R may be used in the management of patients with acute exacerbations of chronic obstructive pulmonary disease (COPD) or patients waiting for lung transplant [1, 2]. As mechanical ventilation in ARDS therapy can lead to ventilator-induced lung injury, ECCO_2_R can be used to enable ultra-protective ventilation in patients without life-threatening hypoxemia [3–6].

Today, effective ECCO_2_R therapy requires blood flow rates of more than 1 L/min and consequently cannulation with relatively large cannulas [1, 3]. Moreover, pure oxygen is frequently used as sweep gas in ECCO_2_R therapy, in order to create a diffusion gradient for CO_2_ to be cleared from the blood [4]. The invasiveness of catheters to generate a sufficient blood flow (BF), as well as mobility limitations due to heavy oxygen bottles, are burdens for potential mobile ECCO_2_R devices [5]. Another limitation of current ECCO_2_R devices is the exclusive permeability of the membrane lung (ML) for gaseous substances and hence the impermeability for dissolved bicarbonate [6]. Since the majority of carbon dioxide is transported as chemically bound HCO_3_^−^, only approximately 10% of the total CO_2_ can pass the ML, necessitating high extracorporeal blood flows to sufficiently deplete the blood of CO_2_ [6].

So far, experimental evaluation of ECCO_2_R setups heavily relies on animal models that are costly and require animal facilities. Considerable efforts to avoid animal experiments are made by both the scientific community and politics. As an alternative, MLs can be tested in mock circuits. Different mock designs have been described in the literature [7–10]. The aim of our study is to present a new mock circuit that reflects the anatomy of a mammal body connected to an ECCO_2_R more closely and to validate that mock in three experimental scenarios.

## Methods

### Composition of the dual-loop circuit

#### Standard experiment protocol

The presented in vitro ECCO_2_R mock consisted of two circuits. The primary circuit was responsible for creating a hypoxic and hypercapnic (venous) environment, which represented the simulated vena cava, while the test ECCO_2_R circuit washed out CO_2_ and oxygenated the venous blood from the primary circuit.

**Fig. 1:**
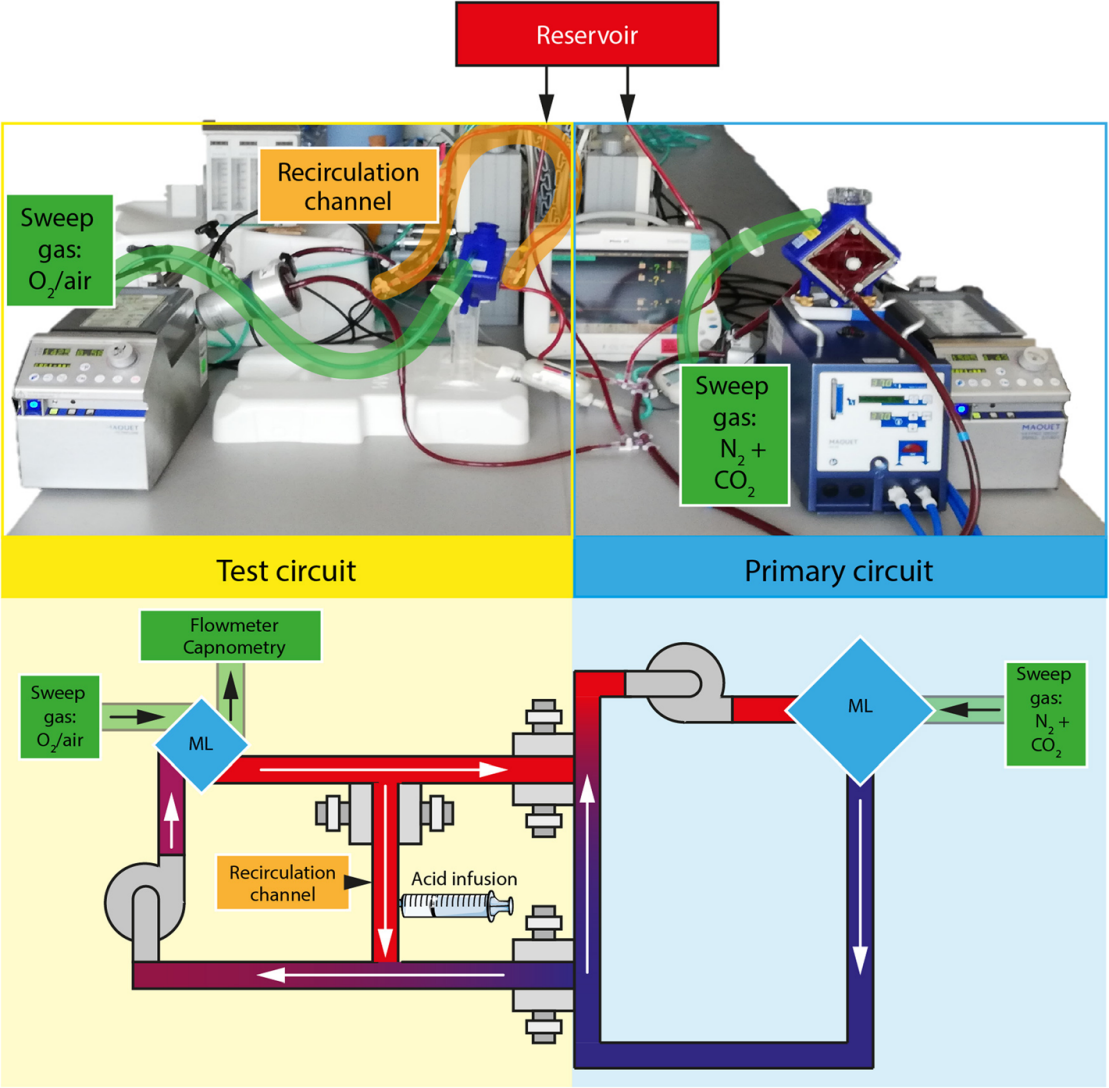
Schematic layout of the mock. The mock consists of two interconnected circuits with one membrane lung (ML) each. The primary circuit represents the simulated organism. It has a blood flow of 5 L/min and creates a hypoxic and hypercapnic (venous) environment with N_2_ and CO_2_ as sweep gases. The actual ECCO_2_R setup to be evaluated is built into the test circuit. CO_2_ removal over the ML in the test setup is quantified. In this work, the influence of different hemoglobin concentrations (12, 7, and 4 g/dL), different sweep gases (O_2_ vs. air), and low-flow scenarios (blood flow amplification with recirculation channel, acidification of recirculated blood) on CO_2_ removal are simulated

The primary circuit consisted of a Getinge PLS (Maquet Cardiopulmonar GmbH, Getinge Group, Rastatt, Germany) circuit composed of a Rotaflow pump, Quadrox membrane lung, and BIOLine® coated PVC tubings. Two luer-lock connectors were installed into the circuit at 10 cm and 30 cm up-stream the centrifugal pump (Fig. 1). Sweep gas of the Quadrox PLS was provided via a gas blender with separate regulation of N_2_, CO_2_, and O_2_ flows. Out of several flow rate combinations a sweep gas flow (${\dot{V}}_{\mathrm{Sweep}}$) of 7.5 L/min N_2_ and 0.55 L/min CO_2_ was chosen to create a venous carbon dioxide partial pressure (*p*_*v*_CO_2_) between 45 mmHg ± 5 mmHg and a venous oxygen saturation of 65% ± 5% (data not shown). Blood flow in the primary circuit was set to 5 L/min. For temperature control of 37 °C, a Maquet HU 35 (Maquet) was used accordingly.

Via the two luer-lock connectors, the test ECCO_2_R circuit was connected to the main circuit. This junction between primary and test circuit could be throttled and simulated the cannulas in this model. The blood flow through these luer-lock connectors was called “cannula flow” in this work. The test circuit used 1/4 PVC tubings, a second Rotaflow centrifugal pump, and a pediatric Getinge Quadrox iD ML as the test ML. A ${\dot{V}}_{\mathrm{Sweep}}$ was applied to the pediatric Quadrox iD ML. At the sweep gas outlet of the ML, a mass flow sensor (TSI41403) and a side-stream capnometer (Philips Intellivue) were installed. Blood flow in the test circuit could be adjusted by closing one of the luer-lock connectors partly or adjusting the rotations per minute on the Rotaflow pump.

As default, ${\dot{V}}_{\mathrm{Sweep}}$ rates of 0.3, 0.5, 0.7, 1, 2, 4, 6, and 7 L/min and blood flows of 0.6 L/min were used in the test circuit. One set of MLs was used per experiment. Each experiment was run for a maximum time of 2 h with one blood filling. A shift of up to two base excess points in 2 h was tolerated.

CO_2_ removal rates (*N* = 6) were calculated from the sweep gas flow rate ${\dot{V}}_{\mathrm{Sweep}}$ at the sweep outlet, the carbon dioxide partial pressure *p*_*g*_CO_2_, and the atmospheric pressure *p*_ges_ according to the following formula:

$$ {\dot{V}}_{{\mathrm{CO}}_2}={\dot{V}}_{\mathrm{Sweep}}\ast \frac{p_g{\mathrm{CO}}_2}{p_{\mathrm{ges}}} $$

#### Choice of test fluid

The mock was filled with either 600 mL of fresh porcine blood or expired human packed red blood cells (PRBC). Porcine blood was added with 10,000 IE Heparin to avoid clotting, 1 g Meropenem to avoid bacterial growth during the measurement, and diluted with a solution of 0.9% saline to a hemoglobin concentration (Hb) of 7 g/dL. As an alternative, PRBC could also be used as test fluid after they were diluted with phosphate-buffered saline (1x) to a Hb of 7 g/dL and adjusted to a pH of 7.20 ± 0.30 with 1000 mmol/L NaHCO_3_ solution. However, in this work, the default test fluid was undiluted porcine blood.

In order to evaluate the two blood fluids, CO_2_ removal rates of fresh porcine blood and human expired PRBC were compared. Blood gas analyses were performed for both circuit fluids and compared. For fresh porcine blood, pH was in range of 7.20 ± 0.30, bicarbonate in the range from 20 to 25 mmol/L, and venous saturation of oxygen 65% ± 5%. In the shown PRBC measurement, the pH was 7.30 bicarbonate 21 mmol/L and venous saturation of oxygen 69%. Lactate was used to assess quality of the blood fluid. For fresh porcine blood, lactate values of up to 10 mmol/L before the measurement were tolerated. For expired PRBC, lactate levels of up to 20 mmol/L the beginning of the measurement series had to be tolerated.

### Evaluation of three experimental ECCO_2_R scenarios

#### Hemoglobin concentration and CO_2_ removal

Three different hemoglobin levels (12, 7, 4 g/dL) were obtained by diluting fresh porcine blood with a solution of 0.9% saline and 24 mmol/L NaHCO_3_. ${\dot{V}}_{\mathrm{Sweep}}$ and blood flow were set according to standard protocol. Blood gas analyses confirmed that bicarbonate was kept constantly at 24 mmol/L ± 3 mmol/L.

#### The air-driven ECCO_2_R

##### Comparison of pure oxygen and air as sweep gases

Pure oxygen or compressed air from gas cylinders were used to create the sweep gas flow. ${\dot{V}}_{\mathrm{Sweep}}$ and BF were set according to standard protocol.

#### The low-flow ECCO_2_R

##### Addition of a recirculation channel to the setup

A recirculation channel with variable throttle was introduced to the test ECCO_2_R circuit according to Fig. 1. A part of the blood behind the test ML was brought back to the pump of the test circuit. Therefore, blood flow through the test ML was higher than the blood flow at the junction between test circuit and primary circuit (i.e., the simulated cannula flow). In this setup, ${\dot{V}}_{\mathrm{Sweep}}$ was kept constant at 5 L/min pure oxygen. The effect on CO_2_ removal of a recirculation channel was compared to the regular circuit by closing the loop fully.

##### Acid-enhanced recirculation

The acid-enhanced recirculation channel was built similar to the recirculation channel described before. An infusion of 0.9% NaCl adjusted to a pH of 1.0 with 25% hydrochloric acid was added via luer-lock to the recirculation channel (Fig. 1). Two different setups were compared. (A) A simulated cannula flow of 260 mL/min, a recirculation flow of 180 mL/min, and an acid infusion rate of 500 mL/h were chosen to evaluate the CO_2_ removal rate of the test ML. (B) The second simulation used a simulated cannula flow of 290 mL/min and a recirculation flow of 170 mL/min and an infusion rate of 1200 ml/h. ${\dot{V}}_{\mathrm{Sweep}}$was kept constantly at 5 L/min for both setups.

### Statistics

Statistical analysis was performed with GraphPad Prism 5.02 (GraphPad Software, Inc., La Jolla, CA). Parametric data was presented as mean ± SD. The Kolmogorov-Smirnov test was used to test for Gaussian distribution. Differences between groups were tested with unpaired *t* test and ANOVA for Gaussian-distributed groups and with the Mann Whitney *U* test and Kruskal-Wallis test for non-Gaussian-distributed groups. *P* values < 0.05 (*) and < 0.01 (**) were considered significant, and *p* values < 0.001 (***) were considered highly significant.

## Results

### Composition of the dual-loop circuit

Using the presented mock circuit model, continuous steady state measurements, with adjustable *p*_*v*_CO_2_ and O_2_ saturation values, were possible.

#### Choice of test fluid

**Fig. 2:**
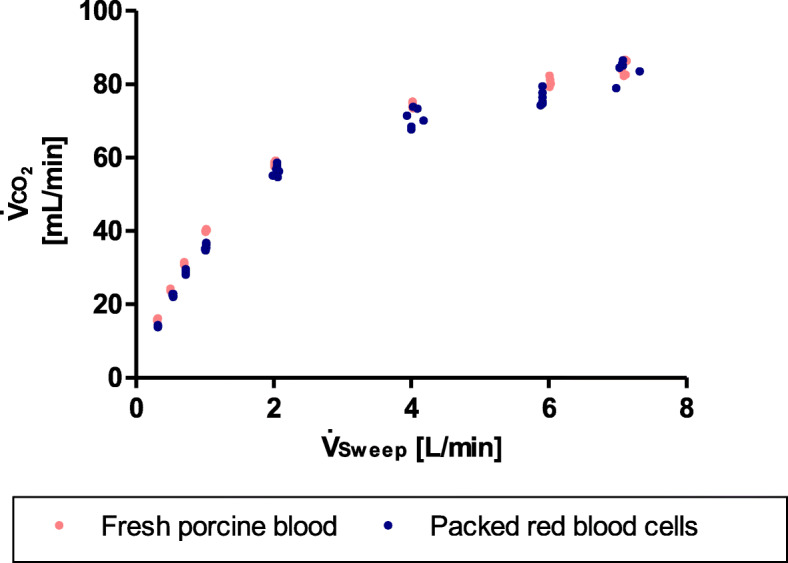
Choice of test fluid. CO_2_ removal rates (${\dot{V}}_{{\mathrm{CO}}_2}$) were similar for fresh porcine blood and diluted packed red blood cells. This measurement was done with a blood flow of 0.6 L/min and O_2_ sweep gas flows (${\dot{V}}_{\mathrm{Sweep}}$) of 0.3, 0.5, 0.7, 1, 2, 4, 6, and 7 L/min in the test circuit

The measurements with expired packed red blood cells showed CO_2_ removal rates comparable to the results with fresh porcine blood diluted to the same hemoglobin concentration (Fig. 2). The non-linear progression of PRBC and porcine blood was similar.

### Evaluation of three experimental ECCO_2_R scenarios

#### Hemoglobin concentration and CO_2_ removal

**Fig. 3:**
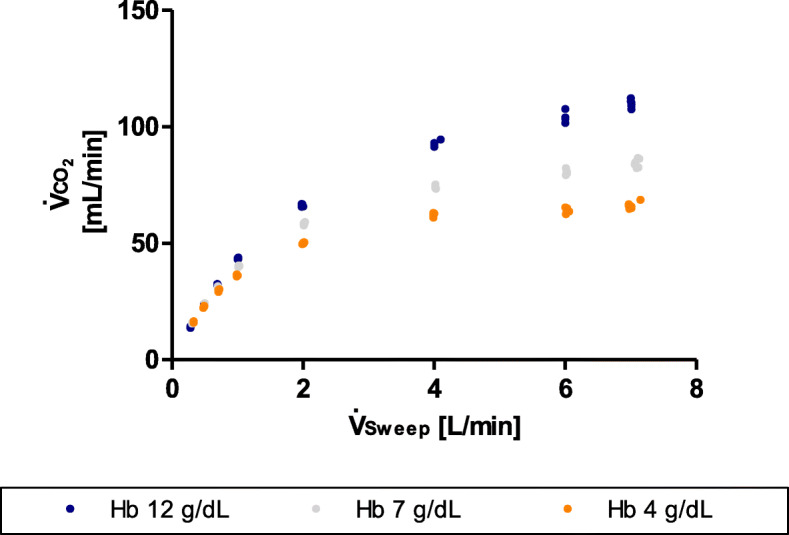
Hemoglobin concentration and CO_2_ removal. Hemoglobin concentrations were set to 12, 7, and 4 g/dL. A blood flow of 0.6 L/min and O_2_ sweep gas flows (${\dot{V}}_{\mathrm{Sweep}}$) of 0.3, 0.5, 0.7, 1, 2, 4, 6, and 7 L/min were used in the test circuit. CO_2_ removal rate was higher for high hemoglobin concentrations (*p* < 0.001)

A strong association of CO_2_ removal rate and hemoglobin concentration was found. The CO_2_ removal of the test ML was higher when a high Hb was used (Fig. 3). The differences in CO_2_ removal between the three Hb values were highly significant (*p* < 0.001). The higher the sweep gas flow rate, the greater was the difference of the three Hb values in CO_2_ removal.

#### The air-driven ECCO_2_R

##### Comparison of pure oxygen and air as sweep gases

Performance of CO_2_ removal (${\dot{V}}_{{\mathrm{CO}}_2}$) showed a plateau at ${\dot{V}}_{\mathrm{Sweep}}$**≥** 6 L/min and above. Below that plateau, CO_2_ removal rate increased with rising ${\dot{V}}_{\mathrm{Sweep}}$ rates, although the measured *p*_*g*_CO_2_ at the sweep gas outlet of the ML was decreasing with rising sweep gas flow rates (Table 1).

**Table 1:** Impact of pure oxygen and air as sweep gases on CO_2_ removal

${\dot{\boldsymbol{V}}}_{\mathbf{Sweep}}$ [L/min]	Sweep gas	***p***_***g***_CO_**2**_ [kPa]	${\dot{\boldsymbol{V}}}_{{\mathbf{CO}}_{\mathbf{2}}}$ [mL/min]	***p***
7	O_2_	1.41	98.92 ± 2.28	*p* < 0.05
7	Air	1.26	89.68 ± 4.06	
6	O_2_	1.56	94.03 ± 0.97	*p* < 0.05
6	Air	1.35	81.37 ± 1.07	
4	O_2_	2.12	85.05 ± 1.04	*p* < 0.05
4	Air	1.77	72.00 ± 1.16	
2	O_2_	3.09	62.51 ± 0.98	*p* < 0.05
2	Air	2.74	54.67 ± 0.64	
1	O_2_	4.05	40.91 ± 1.41	*p* < 0.05
1	Air	3.58	36.14 ± 0.57	
0.7	O_2_	4.35	30.14 ± 0.62	ns
0.7	Air	4.27	29.61 ± 0.55	
0.5	O_2_	4.58	23.29 ± 0.32	*p* < 0.05
0.5	Air	4.12	20.11 ± 0.4	
0.3	O_2_	4.7	13.99 ± 0.25	*p* < 0.05
0.3	Air	4.3	13.13 ± 0.11	

The CO_2_ removal using air was, in most cases, 15–20% less efficient (*p* < 0.05) compared to pure oxygen at the same ${\dot{V}}_{\mathrm{Sweep}}$.

#### The low-flow ECCO_2_R

##### Impact of a recirculation channel on CO_2_ removal

The opening of the channel for recirculation caused an increase of the blood flow through the ML of the test circuit. However, the dynamics in fluid flow between the primary and test circuits as a whole was changed. The pump speed of the test circuit had to be increased to maintain a simulated cannula flow, similar to that before onset of recirculation.

The addition of a recirculation channel in some settings achieved an increased CO_2_ removal rate while in other settings decreased CO_2_ removal rates are observed (Table 2). The reduced CO_2_ removal rates were mainly observed for very high recirculation ratios ($\mathrm{recirculation}\ \mathrm{ratio}=\frac{\mathrm{recirculation}\ \mathrm{flow}}{\mathrm{membrane}\ \mathrm{flow}}\ast 100\%$) relative to the simulated cannula flow, such as 88%.

**Table 2:** Effect of a recirculation channel on CO_2_ elimination

Simulated cannula flow [mL/min]	Recirculation channel flow [mL/min]	Membrane flow [mL/min]	Recirculation ratio [%]	${\dot{\boldsymbol{V}}}_{{\mathbf{CO}}_{\mathbf{2}}}$ [mL/min]	***p***
910	Closed	910		66.67 ± 0.69	*p* < 0.05
900	500	1400	35.7	69.63 ± 0.73	
500	Closed	500		58.31 ± 0.61	ns
500	280	780	35.9	57.97 ± 0.4	
300	Closed	300		48.66 ± 0.46	*p* < 0.05
300	240	540	44.4	49.93 ± 0.43	
200	Closed	200		42.25 ± 0.71	ns
200	140	340	41.2	42.32 ± 0.99	
100	Closed	100		28.76 ± 1.22	*p* < 0.05
100	80	180	44.4	30.93 ± 0.62	
330	Closed	330		55.38 ± 0.88	ns
330	1140	1470	77.6	54.45 ± 1.26	
180	Closed	180		45.16 ± 0.96	*p* < 0.05
180	1310	1490	87.9	41.24 ± 0.43	

##### Impact of HCl infusion on CO_2_ removal

**Fig. 4:**
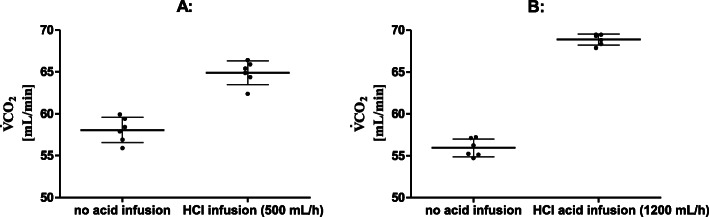
Acidification of recirculated blood. Acidification of blood in the recirculation channel leads to a significantly increased CO_2_ removal. **a** ${\dot{V}}_{\mathrm{Sweep}}$ of 5 L/min, simulated cannula flow of 260 mL/min, recirculation flow of 180 mL/min, and HCl infusion rate of 500 mL/h. **b** ${\dot{V}}_{\mathrm{Sweep}}$ of 5 L/min, simulated cannula flow of 290 mL/min, recirculation flow of 170 mL/min, and HCl infusion rate of 1200 mL/h

The addition of an HCl infusion at 500 mL/h (1200 mL/h) to the recirculation channel with a simulated cannula flow of 260 mL/min (290 mL/min) and a recirculation flow of 180 mL/min (170 mL/min) led to a significant increase in CO_2_ removal rate (Fig. 4). The effect was more pronounced for an acid infusion rate of 1200 mL/h than for 500 mL/h.

## Discussion

### Composition of the dual-loop circuit

The ECCO_2_R mock described in this work shows a reasonable alternative method to gain information about ECCO_2_R and simulate clinical settings. While animal-based ECMO or ECCO_2_R test setups remain gold standard, for certain scientific issues, the animal model might not add additional benefit despite being a lot more complex and costlier. The test circuit in this model was inspired by the Homburg lung, the most commonly used ECCO_2_R platform in our center, which is based on the Rotaflow platform and a pediatric Quadrox iD oxygenator [11]. One advantage of our mock setup in comparison to animal-based ECCO_2_R experiments is the ability to modify blood gas parameters and the hemoglobin level more easily.

Other mock circulation models have been described in the literature that differ however from our approach. Schumer et al. created a closed loop ECMO mock with a single pump, a deoxygenation ML, and the tested oxygenation ML in series [10].

Barret et al. demonstrated a low blood flow single loop ECCO_2_R model and used a Hemolung test ML. The impact of the sweep gas flow (air) on CO_2_ removal and the relationship between ${\dot{V}}_{\mathrm{Sweep}}$ and dead space was investigated [7]. Moreover, Sun et al. tested four commercial MLs in a closed loop circuit with one centrifugal pump [12]. The CO_2_ removal rates for the tested Maquet Quadrox pediatric iD MLs were in the same range as our results using the same ML for a Hb of 15 g/dL (Table 3).

**Table 3:** Comparison of CO_2_ removal rates of the pediatric oxygenator Quadrox iD for different blood flow (BF) and sweep gas flows (${\dot{V}}_{\mathrm{Sweep}}$)

${\dot{V}}_{\mathrm{Sweep}}$	This study (BF 0.6 L/min)	Sun et al. (BF 0.5 L/min) [12]
**1 L/min**	41 mL/min	39 mL/min
**2 L/min**	63 mL/min	61 mL/min
**4 L/min**	85 mL/min	92 mL/min

In contrast to those approaches, our model consists of two interconnected circuits where each circuit has its own centrifugal pump. This allows to create individual blood flows in the primary circuit and the test circuit. Consequently, the physiological blood flow of a mammal body (primary circuit, BF = 5 L/min) connected to an ECCO_2_R device (test circuit) can be demonstrated more precisely. Similar to our approach, de Villiers Hugo et al. have created a dual mock to investigate a low-flow system with a modified dialysis unit [8].

Unlike most of the described mocks, our construction allows to measure continuously [1, 10, 13]. Using N_2_ and CO_2_ as sweep gases on the primary circuit, a constant venous milieu with a *p*_*v*_CO_2_ of 45 ± 5 mmHg can be established throughout the whole measurement series.

#### Limitations of the dual-loop model

However, our study has some limitations. As most experiments were carried out with porcine rather than human blood, the data cannot be directly compared to clinical patients. However, we did evaluate diluted expired human packed red blood cells, although in one exemplary scenario, and found no relevant difference in CO_2_ elimination compared to fresh porcine blood. However, like in most mock circuits, the absence of endothelium and its interaction with the blood limits generalization.

Moreover, the two preclinical scenarios, air-driven and low-flow ECCO_2_R, were measured with undiluted porcine blood samples, as obtained by the butcher, in order to keep already complex setups as simple as possible. Porcine Hb values of 13.5 ± 1.5 g/dL are rather high compared to typical clinical scenarios of patients with hypercapnic respiratory failure. However, a scenario with diluted porcine blood samples was added to consider the association of different hemoglobin concentrations and CO_2_ removal. Not for clinical reasons but to analyze a broad range, the lowest Hb level was set to a rather low 4 g/dL.

#### Choice of test fluid

We conducted this experiment to demonstrate that both fresh porcine blood and diluted expired packed red blood cells qualify for mock circuit fluid with rather similar properties. While we used both fluids in our initial tests with mock circuits, at this point, we prefer fresh porcine blood. Availability of diluted packed red blood cells is limited, as clinical operations only rarely generate expired PRBC. Those few PRBC that are older than 6 weeks have to be discarded and can be redirected to research. Although expiration of PRBC does happen, timing is not predictable, and quality and storage time of those expired packed red blood cells is very inhomogeneous. Moreover, we observed that deoxygenating the system to a sufficient venous environment in the primary circuit was easier when using fresh pig blood. The impact of storage time on biochemical processes of PRBC has been described in literature before.

Prolonged storage of PRBC leads to a left shift of the oxygen dissociation curve and the affinity of oxygen for hemoglobin in the PRBC increases [14, 15]. Moreover, the increase of affinity results in a decrease of 2,3-bisphosphoglycerate concentration in PRBC during storage longer than 2 weeks [16]. Furthermore, a lower pH, due to lactate production by erythrocytes, induces the depletion of 2,3-bisphosphoglycerate and increases the affinity of hemoglobin for O_2_ [15]. As a result, the ability to deoxygenate in the primary circuit is better for fresh porcine blood than for old PRCBs.

In addition, fresh porcine blood samples did not require pH adjustment, as there was very little variance in the samples and the amount of lactate was much lower. Moreover, it was paramount to avoid relevant pH drifts during experiments, and fresh porcine blood was also more stable in this regard.

### Evaluation of three experimental ECCO_2_R scenarios

#### Hemoglobin concentration and CO_2_ removal

The hemoglobin level in the blood has a strong impact on the ability of the test ML to remove CO_2_, as hemoglobin is one of the carriers of CO_2_ in the blood. About 13% of the total CO_2_ amount in the blood is bounded on Hb to carbaminohemoglobin [16, 17]. A lower hemoglobin concentration in the blood reduces the ability to unload CO_2_ from carbaminohemoglobin in exchange for O_2_ at the test ML according to human lungs [18, 19]. Moreover, as described by the Haldane effect, the upload of oxygen to hemoglobin in the test ML induces a release of protons (H^+^) from the hemoglobin molecules [20, 21]. In the blood, there is a reversible equilibrium between bicarbonate and carbon dioxide catalyzed by the enzyme carbonic anhydrase that is stored in erythrocytes [15, 20, 22]. Reducing hemoglobin level, the buffer capacity to provide protons declines as well as the amount of the enzyme carbonic anhydrase to catalyze the reaction. Therefore, less CO_2_ can possibly be eliminated at the test ML.

#### The air-driven ECCO_2_R

In this scenario, various sweep gas flows are tested to find an economical ${\dot{V}}_{\mathrm{Sweep}}$range. While a decrease in oxygen flow by 1 or 2 L/min alone might not seem significant in terms of macroeconomics, it is nevertheless an important step to a more efficient ECCO_2_R design.

As shown in Table 2, for a blood flow of 600 mL/min, a plateau is reached, applying ${\dot{V}}_{\mathrm{Sweep}}$rates ≥ 6 L/min. Therefore, an efficient mobile ECCO_2_R with limited sweep gas supply should stay at the lower sweep gas flow rates of this plateau. Similar results have been shown by Barret et al. for a blood flow of 400 mL/min and a plateau for ${\dot{V}}_{\mathrm{Sweep}}$ ≥ 4 L/min [7].

We propose that the plateau is caused by a decrease of the final *p*_*g*_CO_2_ in the gas phase at the outflow of the test ML, when high ${\dot{V}}_{\mathrm{Sweep}}$ rates are applied. We assume, that using low sweep flow rates, an equilibrium between CO_2_ in gas and in the blood is established early in the hollow fiber of the ML, and the *p*_*g*_CO_2_ determined is similar to the *p*_*v*_CO_2_ in the blood. Increasing ${\dot{V}}_{\mathrm{Sweep}}$, the equilibrium is established subsequently, or the period of contact is too short to reach equilibrium. Therefore, the measured *p*_*g*_CO_2_ of high flow rates is lower than for low flow rates. According to Fick’s principle, a high difference between *p*_*v*_CO_2_ and *p*_*g*_CO_2_ induces a higher diffusion flux, hence a higher CO_2_ elimination. However, high sweep flow rates result in a final *p*_*g*_CO_2_ in the gas phase that is practically zero. A further increase cannot decrease the *p*_*g*_CO_2_ in the gas phase below zero and therefore has minimal effect on the diffusion gradient and the CO_2_ removal rate.

The second aspect of this experiment was the idea to use ambient air as a sweep gas alternative to pure oxygen. This would eliminate the above discussed problem of sweep gas economy altogether. However, as shown in Table 2, CO_2_ removal rate of ambient air is significantly lower than that of pure oxygen. We assume, that this stands in close connection to the Haldane effect as well [21]. Ambient air leads to lower oxygen concentrations in the blood compared to pure oxygen. By uploading less oxygen to hemoglobin, less CO_2_ dissociates from the molecule in exchange [17]. Moreover, a lower pO_2_ in the blood causes a smaller release of protons from hemoglobin and therefore less CO_2_ formation and dissociation [18]. Despite this reduction on CO_2_ removal, we still like the idea of a portable ECCO_2_R with ambient air. A turbine setup, such as used by many CPAP devices, could utilize the infinite supply of ambient air and is most likely less heavy than a gas cylinder. This makes a patient on such a hypothetic ECCO_2_R device independent from pure oxygen and gas cylinders and might promote the mobility of the patient.

#### The low-flow ECCO_2_R

A major problem of low-flow cannulas, such as dialysis catheters, is their inability to create a sufficient BF across the ML, leading to an increased risk of ML clotting. A previous study has shown that there is a correlation of areas with low flow speed and thrombus formation in membrane oxygenators [23]. One solution for this problem is to increase flow speed in the membrane lungs by using smaller oxygenators, with smaller cross sections. However, in our opinion it is preferable to use commonly available oxygenators with higher surface area. Therefore, another approach is the use of a recirculation channel, a trick to recirculate the limited blood volume from the cannula more than once before sending it back into the patient. However, only flow volume is increased by such a recirculation channel, not the CO_2_ content, because the recirculated blood was already CO_2_ depleted during the first passage through the ML. To further increase CO_2_ removal from the mixed blood, HCO_3_^−^ can be converted into membrane diffusible CO_2_ with the addition of acid.

##### Impact of a recirculation channel on CO_2_ elimination

When first evaluating recirculation channels, an obviously reduced CO_2_ removal compared to an otherwise identical setup without a recirculation channel was noticeable. Pump rotation speed was kept constant when opening the loop inducing a lower simulated cannula flow (data not shown).

However, for the comparisons shown in this paper, we decided that the simulated cannula flow, which defines the amount of CO_2_-rich blood that goes into the test setup, should be held constant. With matched simulated cannula flows, the overall trend for lower CO_2_ removal rates with recirculation channels is gone, albeit at the expense of higher pump rotation speeds.

Small and large recirculation ratios vary CO_2_ removal efficiency because of a different CO_2_ content in the mixed blood and a different total membrane flow. Increasing the recirculation ratio decreases the carbon dioxide partial pressure in the mixed blood (*p*_*m*_CO_2_) hence the transmembrane gradient, but on the other hand, increases flow speed in the ML, two factors with inverse influence on CO_2_ removal rate. With ECMO flow rates, rather than ECCO_2_R flow rates in this work, Madhani et al. also found an increased CO_2_ transfer rate for blood recirculation [24]. However, in his setup, a cannula flow of 3.5 L/min and a recirculation flow of 6.5 L/min were used. This recirculation ratio of 65% is above our efficiency increasing ratios but below those recirculation ratios, where our setup showed a decrease in efficiency. We suggest that there is an optimal recirculation ratio, somewhere between 50 and 75%. The evaluation of this optimum should be subject of future research.

##### Impact of acidification of the recirculated blood

Achieving increased CO_2_ removal by local acidification has been described in previous studies [2, 5, 6, 9, 25–27]. However, this is the first study to combine an acidification step with a recirculation channel. The acidification can be used to overcompensate slight disadvantages in the CO_2_ washout of recirculation channels with high recirculation ratio. The acid transforms bicarbonate (effectively chemically dissolved CO_2_) into physically dissolved CO_2_, hence restoring the *p*_*m*_CO_2_ level in the recirculated blood and increasing CO_2_ removal in the ML. In our study, the strong inorganic acid HCl was used, as Cl^−^ has little metabolic effects. However, like previous studies, we also identified hemolysis caused by both the acid and mechanical blood trauma in the bends of the recirculation channel as major problem that needs to be solved before this approach can be used in clinical studies [25].

## Conclusion

Our ECCO_2_R mock represents a novel method to generate data with animal free experiments as it simulates a mammal body connected to an ECCO_2_R. As a practical consequence of this work, we suggest that a gas cylinder free and mobile ECCO_2_R could use air as sweep gas with only moderate reduction in efficiency. Moreover, we conclude that recirculation channels and acid enhancement are promising tools to improve ECCO_2_R therapy that should be topic of future research.

All in all, our model is an improvement of so far established mock circuits, and more ECCO_2_R setups will be tested in the future.

## Data Availability

The datasets used and/or analyzed during the current study are available from the corresponding author on reasonable request.

## Abbreviations

ECCO_2_R: Extra corporal carbon dioxide removal
CO_2_: Carbon dioxide
Hb: Hemoglobin
ML: Membrane lung
${\dot{V}}_{\mathrm{Sweep}}$: Gas flow rate
*p*_*g*_CO_2_: Carbon dioxide partial pressure in gas phase
*p*_*v*_CO_2_: Carbon dioxide partial pressure in venous blood
*p*_*m*_CO_2_: Carbon dioxide partial pressure in mixed blood
p_ges_: Atmospheric pressure
BF: Blood flow
ARDS: Acute respiratory distress syndrome
COPD: Chronic obstructive pulmonary disease
PVC: Polyvinyl chloride
PRBC: Packed red blood cells
${\dot{V}}_{CO_2}$: CO_2_ removal rate
HCL: Hydrochloric acid

## References

[1] Jeffries RG, Lund L, Frankowski B, Federspiel WJ (2017). An extracorporeal carbon dioxide removal (ECCO2R) device operating at hemodialysis blood flow rates. Intensive Care Med Exp.

[2] Zanella A, Mangili P, Giani M (2014). Extracorporeal carbon dioxide removal through ventilation of acidified dialysate: an experimental study. J Heart Lung Transplant.

[3] Hilty MP, Riva T, Cottini SR (2017). Low flow veno-venous extracorporeal CO_2_ removal for acute hypercapnic respiratory failure. Minerva Anestesiol.

[4] Morelli A, Del Sorbo L, Pesenti A (2017). Extracorporeal carbon dioxide removal (ECCO2R) in patients with acute respiratory failure. Intensive Care Med.

[5] Scaravill V, Batchinsky AI (2016). Extracorporeal carbon dioxide removal enhanced by lactic acid infusion in spontaneously breathing conscious sheep. Crit Care Med.

[6] Zanella A, Giani M, Redaelli S (2013). Infusion of 2.5 meq/min of lactic acid minimally increases CO_2_ production compared to an isocaloric glucose infusion in healthy anesthetized, mechanically ventilated pigs. Crit Care Lond Engl.

[7] Barrett NA, Hart N, Camporota L (2020). In-vitro performance of a low flow extracorporeal carbon dioxide removal circuit. Perfusion.

[8] de Villiers HJ, Sharma AS, Ahmed U, Weerwind PW (2017). Quantification of carbon dioxide removal at low sweep gas and blood flows. J Extra Corpor Technol.

[9] Arazawa DT, Kimmel JD, Finn MC, Federspiel WJ (2015). Acidic sweep gas with carbonic anhydrase coated hollow fiber membranes synergistically accelerates CO2 removal from blood. Acta Biomater.

[10] Schumer E, Höffler K, Kuehn C (2018). In-vitro evaluation of limitations and possibilities for the future use of intracorporeal gas exchangers placed in the upper lobe position. J Artif Organs.

[11] Seiler F, Trudzinski FC, Hennemann K (2017). The Homburg lung: efficacy and safety of a minimal-invasive pump-driven device for veno-venous extracorporeal carbon dioxide removal. J Am Soc Artif Intern Organs.

[12] Sun L, Kaesler A, Fernando P (2018). CO_2_ clearance by membrane lungs. Perfusion.

[13] May AG, Jeffries RG, Frankowski BJ (2018). Bench validation of a compact low-flow CO_2_ removal device. Intensive Care Med Exp.

[14] Valtis DJ (1954). Defective gas-transport function of stored red blood-cells. Lancet Lond Engl.

[15] Högman CF, Löf H, Meryman HT (2006). Storage of red blood cells with improved maintenance of 2,3-bisphosphoglycerate. Transfusion (Paris).

[16] Chanutin A, Curnish RR (1967). Effect of organic and inorganic phosphates on the oxygen equilibrium of human erythrocytes. Arch Biochem Biophys.

[17] Dash RK, Bassingthwaighte JB (2010). Erratum to: Blood HbO2 and HbCO2 dissociation curves at varied O2, CO2, pH, 2,3-DPG and temperature levels. Ann Biomed Eng.

[18] Klocke RA (1988). Velocity of CO_2_ exchange in blood. Annu Rev Physiol.

[19] Hsia CCW (1998). Respiratory function of hemoglobin. N Engl J Med.

[20] Hill E, Power G, Longo L (1973). Mathematical simulation of pulmonary O_2_ and CO_2_ exchange. Am J Physiol-Leg Content.

[21] Siggaard-Andersen O, Garby L (1973). The Bohr effect and the Haldane effect. Scand J Clin Lab Invest.

[22] Lindskog S (1997). Structure and mechanism of carbonic anhydrase. Pharmacol Ther.

[23] Funakubo A, Taga I, McGillicuddy JW (2003). Flow vectorial analysis in an artificial implantable lung. J Am Soc Artif Intern Organs.

[24] Madhani SP, May AG, Frankowski BJ et al (2019) Blood recirculation enhances oxygenation efficiency of artificial lungs. J Am Soc Artif Intern Organs. 10.1097/MAT.0000000000001030 10.1097/MAT.0000000000001030 31335366

[25] Zanella A, Patroniti N, Isgrò S (2009). Blood acidification enhances carbon dioxide removal of membrane lung: an experimental study. Intensive Care Med.

[26] Takahashi N, Nakada T-A, Oda S (2018). Efficient CO_2_ removal using extracorporeal lung and renal assist device. J Artif Organs.

[27] Scaravilli V, Kreyer S, Linden K (2015). Enhanced extracorporeal CO_2_ removal by regional blood acidification: effect of infusion of three metabolizable acids. J Am Soc Artif Intern Organs.

